# Targeted literature review to understand the patient experience of essential thrombocythemia and evaluate existing clinical outcome assessment measures

**DOI:** 10.1186/s41687-026-01048-7

**Published:** 2026-10-01

**Authors:** Amy Howerter, Xiaoqin Yang, Kelly McQuarrie, Lucy Lloyd-Price, Joseph Gyorkey, Stacie Hudgens

**Affiliations:** 1 Clinical Outcomes Solutions, Tucson, AZ USA; 2 Merck & Co., Inc., Rahway, NJ USA; 3 Clinical Outcomes Solutions, Folkestone, Kent UK

## Abstract

**Background:**

The use of Clinical Outcome Assessments (COA) instruments in blood cancers such as myeloproliferative neoplasms (MPN) is widely documented, however little is known about Essential Thrombocythemia (ET). A targeted literature review was conducted to identify the concepts of interest to the ET population and to develop a conceptual framework. Using this framework, identification and review of COA instruments will help determine if there are existing instruments fit for purpose for use in ET studies.

**Methodology:**

Targeted reviews in May and October 2023 synthesized the literature on ET symptoms and impacts to derive a conceptual model and identify suitable COAs. Eligible articles consisted of ET only populations and extended to MPN samples that included ET patients. A gap analysis was conducted on selected instruments.

**Results:**

Of the 890 abstracts reviewed, 206 were included in the analysis. An ET conceptual model that encompassed disease-defining concepts (fatigue most common), proximal, distal, and general life impacts was created. MPN specific measures such as the Myelofibrosis Symptom Assessment Form v4.0 (MFSAF), and Myeloproliferative Neoplasm Symptom Assessment Form Total Symptom Score (MPN-SAF TSS; MPN-10), European Organization for Research and Treatment of Cancer Quality of Life Questionnaire-Core Questionnaire (EORTC QLQ-C30), Patient-Reported Outcomes Measurement Information System (PROMIS) - Fatigue, and Brief Fatigue Inventory (BFI) were deemed the most appropriate for the gap analysis. Each had strengths and limitations related to conceptual coverage, evidence for measurement properties, and feasibility for use in clinical trials.

**Conclusions:**

The MFSAF v4.0, PROMIS-Fatigue, and EORTC QLQ-C30 are considered appropriate for use in deriving trial endpoints in ET; however further work is needed to assess the fitness-for-purpose in the ET population as most validation studies were performed in mixed MPN or oncology samples.

**Supplementary information:**

The online version contains supplementary material available at https://doi.org/10.1186/s41687-026-01048-7.

## Background

Essential thrombocythemia (ET) is a rare blood neoplasm, a subtype of myeloproliferative neoplasms (MPN), characterized by clonal blood cell proliferation and excessive production of platelets [1]. Patients with ET are at risk for experiencing bleeding and thrombotic complications and disease progression to myelofibrosis and acute myeloid leukemia. Patients with ET experience debilitating symptoms including night sweats, bone pain, abdominal discomfort, fatigue, fever, and weight loss, which can significantly impact their quality of life [2, 3].

As therapies are being developed for ET, it is important to have fit-for-purpose instruments to include in clinical trials to measure the ET patient experience. While there are instruments developed for the MPN population, there are no instruments developed specifically for the ET specific patient experience.

The purpose of the qualitative and descriptive study literature review was to generate insights of the core and associated symptoms of ET and how these symptoms impact patients and their families. This helped to identify what is important and relevant to patients with ET and gain a conceptual understanding of their experiences to support the development of a patient-centric conceptual model. This formed the foundation from which COAs identified as potentially having relevance for use in ET are evaluated for ET conceptual comprehensiveness. The key objectives of the targeted literature review were to identify: 1) ET-specific proximal and distal symptoms and impacts to inform a patient-centric conceptual model of the ET patient experience; 2) COA measures that assess each of the proximal disease-related symptoms and impacts of ET; 3) Map the proximal disease-defining concepts in the ET conceptual model to each of the identified COA measures; and 4) Conduct a gap analysis summarizing the development and psychometric evaluation of identified candidate measures and the extent to which the measures have been utilized in ET clinical trials.

## Methods

### Search strategy

**Fig. 1:**
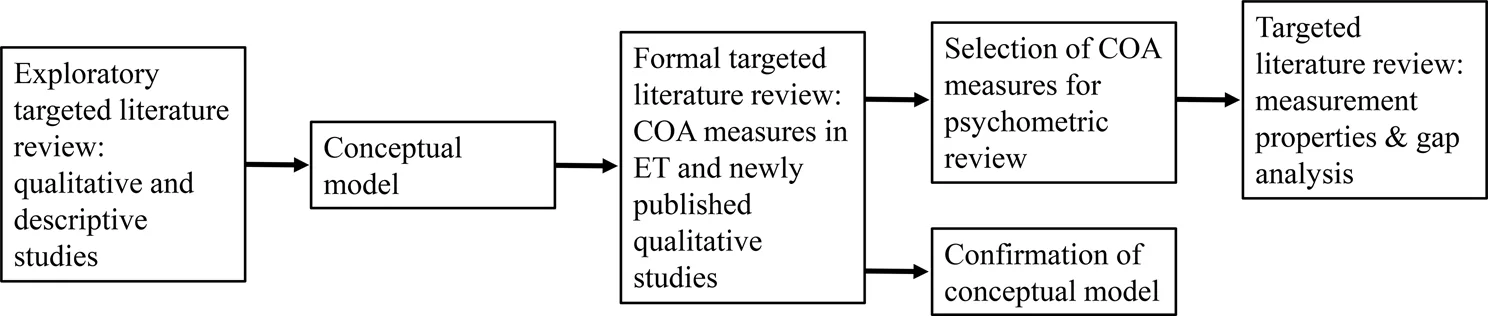
Phased methodology for ET targeted literature review

To address the study objectives, two targeted literature reviews were conducted (Fig. 1). The search terms for the published literature in the Ovid databases is provided in the Supplementary Files. An initial exploratory search was conducted to identify qualitative data and descriptive studies conducted in the ET population to guide a conceptual model (Table A, supplementary material). Although there is typically a focus on qualitative studies only when building a conceptual model, the focus of this exploratory search was widened to include descriptive studies from either clinical trial or observational cohorts to supplement the lack of qualitative data. The search, therefore, included studies from qualitative, observational, prospective, epidemiological, and clinical studies that could potentially provide descriptions of symptoms and impacts of ET and further extended to patient informed publications like the MPN Voice of the Patient report and the Patients Like Me website.

A second structured search was then conducted to identify COA measures and evidence of measurement properties conducted in ET populations including any additional qualitative literature studies published since the earlier search or not previously identified (Table B of Supplementary material). Studies including MPN or disorders where ET or post-ET myelofibrosis (MF) patients were a primary or subset cohort were included in both reviews. The phased methodological approach used for this targeted literature review is summarized in Fig. 1, and utilized publications from 2012 to 2023.

Literature searches were conducted across a variety of platforms to ensure a systematic approach in the collection of all relevant published information. The EMBASE, Medline, and PsycINFO databases were searched via Ovid, a medical research platform. Searches were conducted across all 3 databases together, and duplicates removed automatically. Alternative terms within a particular category were combined using the Boolean term OR, and the different categories were combined using the Boolean term AND. The adjacent operator (ADJ) was used to identify literature where 2 terms appeared near each other (e.g., “self ADJ1 rated” searched for both terms next to each other in either order, “self ADJ2 questionnaire” searched for both terms and up to 1 word in between them.

Date limitations for the structured search were the last 10 years (2012 to October 2023) to capture the latest and most relevant information related to ET, additional limitations were “human” and “English.” Searches were restricted to use of terms in the title, abstract, or keywords sections of the publications.

Additionally, PROQOLID™, a comprehensive online database provided by ePROVIDE™, was searched for COA measures listed under the therapeutic area of “myeloproliferative” to ensure that sufficient detail is provided to support the ET indication. Any additional COA measures not identified in the Ovid search were collated and provided from the literature and relevant gray literature sources.

Eligible articles included ET-only populations and extended to MPN samples including ET and post-ET MF; qualitative results or descriptive studies; and COA information.

### Study selection

A staged review process of the initial search results allowed for a targeted approach to identify and select relevant literature for review. An experienced researcher performed a first pass review of the title and abstract of all medical literature identified from Ovid to eliminate irrelevant articles from the search. The full content of relevant papers was then reviewed. If a paper’s relevance was unclear from the abstract (or if the abstract was not available), the full paper was requested to assess eligibility. A second pass review then further refined the literature based on the approved inclusion/exclusion criteria. Disputes as to eligibility were referred to a third party on the research team.

Articles selected for full text review were evaluated and salient information pertaining to the study aim, sample demographics, methodology, and results of each article were summarized. From the extracted information, key results were identified relating to the experiences of patients. The model provided a visual representation of the most important and relevant concepts and domains for the target population.

## Results

### Conceptual model

An Ovid qualitative and descriptive study literature review identified 215 publications; it yielded 1 publication describing qualitative research with post-ET MF patients [4] and 4 observational study publications presenting ET patient data derived from 2 large MPN studies, the Myelofibrosis and Essential Thrombocythemia Observational Study (MOST) [1, 3] and the MPN Landmark Survey. [5–7] One additional qualitative study was revealed during the second phase literature search also provided input into the conceptual model [8]. Information gleaned from these studies, together with the data from the MPN Voice of the Patient report [9] and the Patients Like Me [10] website, informed the development of a conceptual model (see Supplementary Table C for details). The MPN Voice of the Patient report, a summary of an FDA led patient focused drug development public meeting provided valuable insights into patient reported symptoms and impacts. Similarly, the Patients Like Me website allows patients to record their symptom experience.

**Fig. 2:**
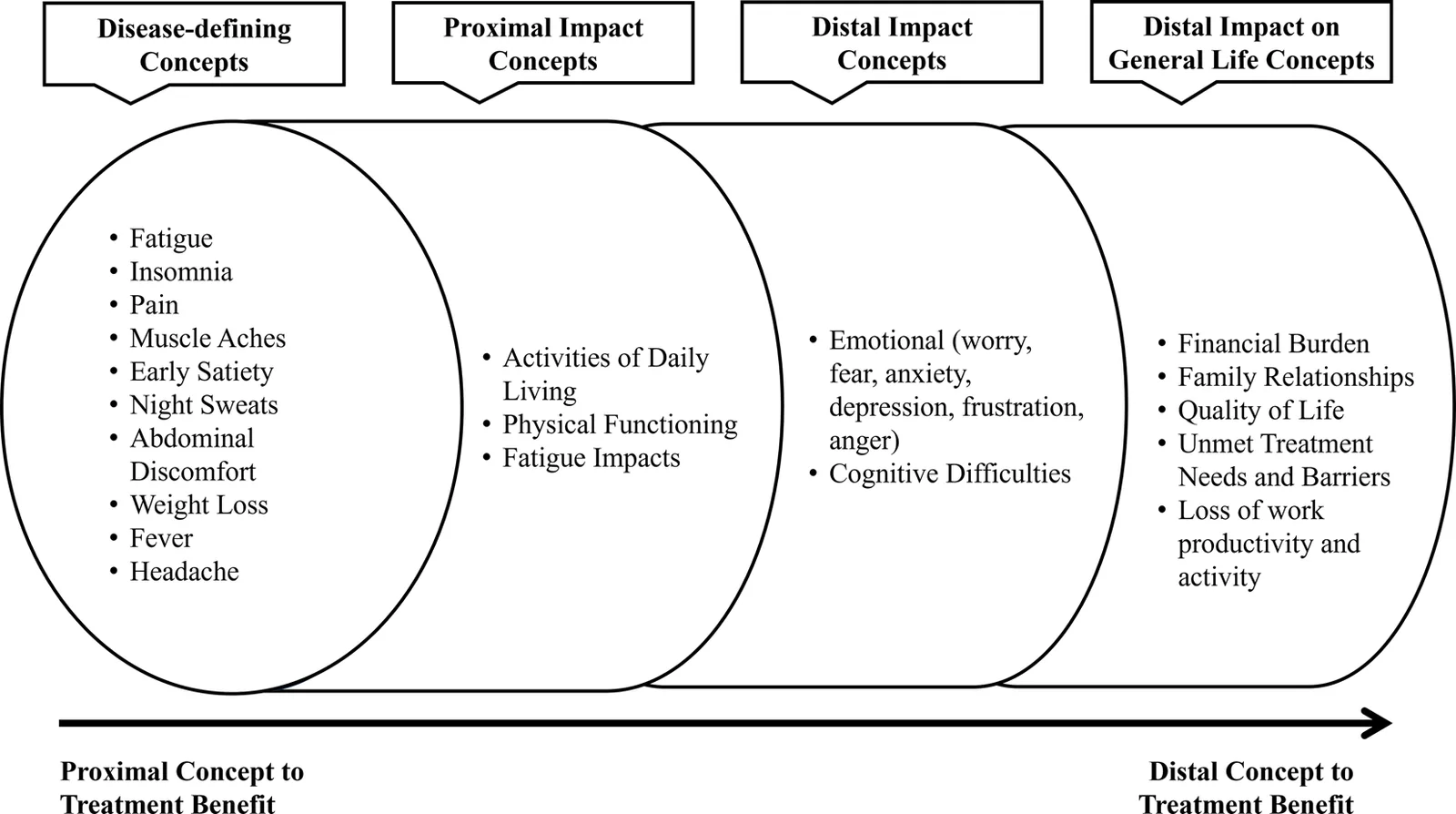
Conceptual model of ET patient experience

The conceptual model (Fig. 2) encompasses ET disease-defining concepts (10 symptoms, fatigue most prevalent), proximal impacts (physical functioning, fatigue impacts, etc), distal impacts (emotional, cognitive), and distal/general life impacts (financial, relationships, etc). [11] These patient centric symptoms and impacts of ET identified from the literature review were reviewed, analyzed, and applied to develop a literature informed conceptual model of ET. The model is organized along a continuum of proximal (directly affecting health) to distal (indirectly affecting health) concepts for treatment benefit. Ten disease-defining concepts and 9 impacts (3 proximal disease impact concepts, 2 distal disease impact concepts and 4 distal impact on general life concepts) were identified. Fatigue was consistently reported as a key symptom from each of the literature sources (see Supplemental Table C). Other reported symptoms of insomnia, pain, muscle aches, early satiety, night sweats, abdominal discomfort, weight loss and fever were included in the disease-defining symptoms concept. Proximal disease impacts included activities of daily living, physical functioning and impacts of fatigue. Distal disease impacts, or those that indirectly affect HRQoL, were emotional distress (worry, fear, anxiety, depression, frustration and anger) and cognitive difficulties. Financial burden, family relationships, quality of life, and unmet treatment needs and barriers were presented as more distal, general life impacts. Organizing symptoms and impacts along the proximal-distal concepts to treatment benefit continuum helped distinguish what symptoms or impacts may have benefited most from a treatment intervention and guided selection of COAs for an endpoint strategy (Fig. 2).

### COA identification

**Fig. 3:**
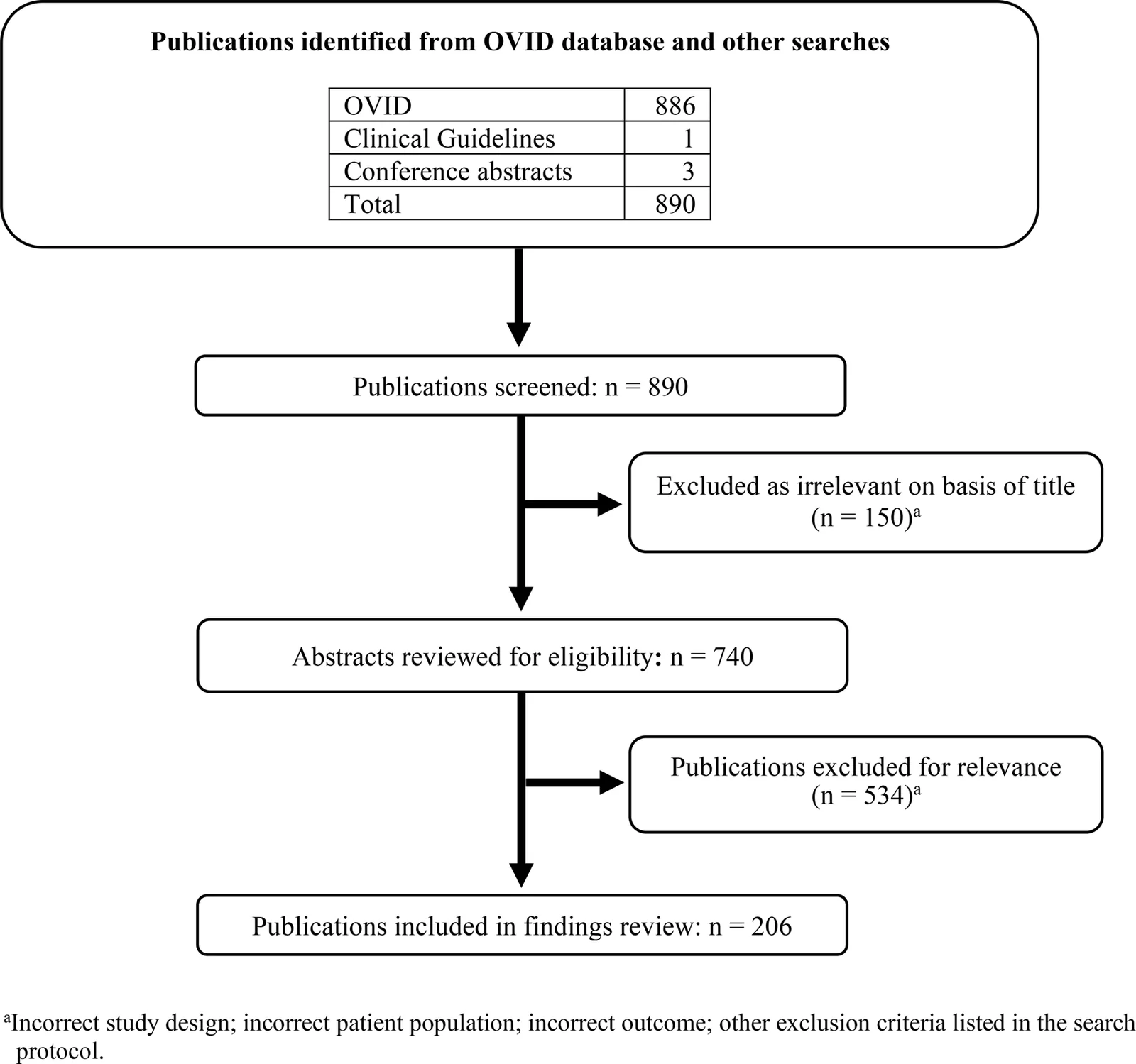
Ovid database search findings

The second targeted literature review focusing on COA measures using Ovid, PROQOLID, and gray literature searches combined resulted in 890 initial publications of which 886 were from Ovid, 1 a clinical guideline, and 3 conference abstracts from ISPOR, ISOQOL and ASH. Publications were classified into 5 general categories: 1) RCT, Clinical controlled trial, Qualitative; 2) Prospective, Observational; 3) Meta Analysis; 4) Systematic Review; and 5) Unrelated study design or population mismatch, which could include non-human studies, incorrect patient population, no COA was used, or other pre-specified exclusion criteria. An initial review based on title alone excluded 150 publications (category 5) leaving 740 for the detailed abstract review. Following abstract screening and review of full text, if available, an additional 534 were excluded (category 5) leaving a total of 206 eligible publications for detailed reporting. The flow diagram from the Ovid and gray literature searches are detailed in Fig. 3.

The COAs identified (>40) in the literature review were primarily patient-reported outcome (PRO) measures. The list of PROs was separated into 5 conceptual categories: MPN symptom specific (*n* = 9); generic health-related quality of life (HRQoL) measures (*n* = 12); fatigue specific (*n* = 6); pain specific (*n* = 2); and, psychological impact focused (*n* = 8). Five PROs were selected for further consideration for use in ET research by examining the frequency of publication in the target population, whether it was designed specifically for ET patients, and whether it had precedence of use in clinical trials for the same/similar endpoints for ET or other MPN conditions.

The 5 selected PROs that were taken forward to the next stage of review were the Myelofibrosis Symptom Assessment Form (MFSAF) v4.0 [12], the Myeloproliferative Neoplasm Symptom Assessment Form Total Symptom Score (MPN-SAF TSS; MPN-10) [13], the European Organization for Research and Treatment of Cancer Quality of Life Questionnaire-Core Questionnaire (EORTC QLQ-C30) [14], the Patient-Reported Outcomes Measurement Information System (PROMIS) – Fatigue [15, 16], and the Brief Fatigue Inventory (BFI) [17]. Table 1 provides descriptive details for each instrument.

**Table 1:** Instrument details for the 5 selected PROs for Gap analysis

Instrument	Instructions	Items/Domains	Response Options	Recall Period
MFSAF v4.0	During the past 24 hours (or past 7 days), how severe was your worst	Fatigue (weariness, tiredness) Night sweats (or feeling hot or flushed) itching Abdominal discomfort (feeling pressure or bloating) Pain under your ribs on your left side Feeling of fullness you had after beginning to eat Bone pain (not joint or arthritis pain)	0 (Absent) to 10 (Worst Imaginable)	Past 24 hours or past 7 days
MPN-SAF TSS; MPN-10^a^	Please rate your fatigue (weariness, tiredness) by selecting the number that best describes your WORST level of fatigue during the past 24 hours 1 to 10 (0 if absent) ranking 1 is most favorable and 10 least favorable	Worst level of fatigue	0 (No fatigue) to 10 (Worst imaginable)	Past 24 hours
	Select the number that best describes, during the past week, how much difficulty you have had with each of the following symptoms.	Early satiety Abdominal Pain Inactivity Concentration problems Night sweats Itching (pruritus) Bone pain Fever > 37.8◦C or >100◦F Weight loss	0 (Absent) to 10 (Worst Imaginable)	During the past week
PROMIS-Fatigue 7a	In the past 7 days, how often did you(r)	Feel tired Experience extreme exhaustion Run out of energy Fatigue limit you at work (include work at home) Too tired to think clearly Too tired to take a bath or shower Have enough energy to exercise strenuously	1 – Never 2 – Rarely 3 – Sometimes 4 – Often 5 – Always	In the past 7 days
EORTC-QLQ-C30		Domains: Global health status/QoL Functional scales: physical, role, emotional, cognitive, social Symptom scales/items: Fatigue, Nausea and vomiting, Pain, Dyspnea, Insomnia, Appetite loss, Constipation, Diarrhea, Financial difficulties	1 – Not at all 2 – A little 3 – Quite a bit 4 – Very Much	During the past week
BFI	Please rate your fatigue (weariness, tiredness) by circling the one number that best describes your	Fatigue right NOW USUAL level fatigue WORST level of fatigue	0 (No Fatigue) to 10 (As bad as you can imagine)	Right NOW During the past 24 hours
	Circle the one number that describes how, during the past 24 hours, fatigue has interfered with your	General activity Mood Walking ability Normal work (includes both work outside the home and daily chores) Relations with other people Enjoyment of life	0 (Does not interfere) to 10 (Completely interferes)	During the past 24 hours

BFI = Brief Fatigue Inventory; EORTC QLQ-C30 = European Organization for Research and Treatment of Cancer Quality of Life Questionnaire-Core Questionnaire; MFSAF = Myelofibrosis Symptom Assessment Form; MPN = myeloproliferative neoplasms; PROMIS Fatigue 7a = PROMIS Fatigue 7-item adult v1.0

^a^ Naming of the MPN-SAF can vary in publications as the MPN-SAF, MPN-SAF TSS, or MPN-10. We use the MPN-SAF TSS; MPN-10 notation throughout this publication

### Concept mapping

Mapping the 5 PROs with the ET conceptual model revealed the MFSAF 4.0 covers 5 disease defining concepts of ET (fatigue, pain, early satiety, night sweats, and abdominal discomfort) and no impacts. The MPN-SAF TSS; MPN-10 covers 7 disease-defining concepts of ET (fatigue, pain, early satiety, night sweats, abdominal discomfort, weight loss, and fever) as well as impacts of physical functioning and cognitive difficulties. The PROMIS-Fatigue 7a and BFI only cover fatigue and impact of fatigue concepts while the EORTC QLQ-C30 covers some disease concepts in addition to proximal and distal disease impacts and impacts on general life. Table 2 provides an indication of conceptual coverage from an item or domain score for each of the 5 PRO measures reviewed.

**Table 2:** PRO concept coverage – overview

Disease-defining Concepts from the Conceptual Model	MFSAF v4.0	MPN-10	EORTC QLQ-C30	PROMIS Fatigue 7a	BFI
Fatigue	✔	✔	✔	✔	✔
Insomnia	X	X	✔	X	X
Pain	✔ (bone pain and pain under ribs on left side)	✔ (bone pain)	✔ (non-specific)	X	X
Muscle Aches	X	X	X	X	X
Early Satiety	✔	✔	X	X	X
Night Sweats	✔	✔	X	X	X
Abdominal Discomfort	✔	✔	✔	X	X
Weight Loss	X	✔	X	X	X
Fever	X	✔	X	X	X
Headache	X	X	X	X	X
**Proximal Impact Concepts**					
Activities of Daily Living	X	X	✔	X	X
Physical Functioning	X	✔ (inactivity)	✔	X	X
Fatigue Impacts	X	X	✔	✔	✔
**Distal Impact Concepts**					
Emotional (worry, fear, anxiety, depression, frustration, anger)	X	X	✔	X	X
Cognitive Difficulties	X	✔ (concentration problems)	✔	X	X
**Distal Impact on General Life Concepts**					
Financial Burden	X	X	✔	X	X
Family Relationships	X	X	✔	X	X
Quality of Life	X	X	✔	X	X

BFI = Brief Fatigue Inventory; EORTC QLQ-C30 = European Organization for Research and Treatment of Cancer Quality of Life Questionnaire-Core Questionnaire; MFSAF = Myelofibrosis Symptom Assessment Form; MPN = myeloproliferative neoplasms; PROMIS Fatigue 7a = PROMIS Fatigue 7-item adult v1.0

Key:✔ = concept is present in an item or domain score; X = concept not present in the measure

### Gap analysis

A gap analysis was conducted to review and identify gaps in the context of current regulatory standards for assessing fit-for-purpose instruments to be used in clinical trials [18, 19]. Components of psychometric validation are displayed in Table 3 for each measure. No studies reported psychometrics specific to an ET sample, however several studies used MPN samples that included ET patients. Symbols in the table reflect whether validation included MPN or MF/oncology samples only. Four of the 5 measures had published articles assessing content validity although utilization of qualitative methods with patient interviews and instrument debriefing was limited in the MPN population. Psychometric support for each of the measures was generally strong, but specific validation in MPN or ET samples for responsiveness and meaningful change thresholds is needed.

**Table 3:** Overview of measurement properties for reviewed measures

Measure	Content Validity	Internal Consistency	Test-Retest Reliability	Convergent or Divergent Validity	Known Groups Validity	Responsiveness	Meaningful Change
MFSAF v4.0 [20–22]	O	✔	✔	✔	✔	☑	X
MPN-SAF TSS; MPN-10 [13, 20, 23]	O	✔	✔	✔	✔	✔	X
PROMIS Fatigue 7a [24–26]	X	☑ [26]	O [26]	O [26]	O [26]	O [24]	O [25]
EORTC-QLQ-C30 [14, 24, 27]	O [27]	☑ [14]	☑	☑ [14, 27]	☑ [14]	☑ [24]	X
Brief Fatigue Inventory [28, 29]	X	☑ [28, 29]	O [28, 29]	☑ [28, 29]	X	O	O

EORTC QLQ-C30 = European Organization for Research and Treatment of Cancer Quality of Life Questionnaire-Core Questionnaire; MFSAF = Myelofibrosis Symptom Assessment Form; MPN = myeloproliferative neoplasms; PROMIS Fatigue 7a = PROMIS Fatigue 7-item adult v1.0

Key: ✔ = good evidence present in MPN; ☑=good evidence present in MF only or other Oncology; O = limited amount of evidence available in MPN; X = no supporting evidence identified

### MFSAF v4.0

The face validity of the MFSAF v4.0 for use as an endpoint is strong and initial development involved participants with MPN including ET (ref). The instructions and response options are clear and consistent for each item, and the recall period is suitable (either 24 hours or 7 day versions are available). The MFSAF v4.0 has been psychometrically validated using data from 513 subjects with essential thrombocythemia (37%), polycythemia vera (41%), and MF (22%) [20]. Results were reported overall and for an MF sub-sample, but not reported for the ET sub-sample separately. Using the overall MPN data, internal consistency of the measure was strong in the overall sample (Cronbach’s α = 0.86). Intraclass correlations showed strong test-retest reliability in the overall sample (intraclass correlation coefficient [ICC] = 0.98, 95% CI: 0.97 to 0.98). The MFSAF v4.0 showed strong correlations with related measures including its earlier version the MFSAF v2.0 TSS (overall sample: *r* = 0.96) and the similar MPN-SAF TSS; MPN-10 (overall *r* = 0.92), supporting convergent validity. Discriminant (known groups) validity was also demonstrated, with the measure showing statistically significant differences in scores among subjects with and without enlarged spleen (*p* < 0.001) in two publications [20, 21]. The MFSAF v4.0 predecessor, the MFSAF v2.0, has shown the ability to detect change in clinical studies in MF which included post-ET samples [24], but no study has published a meaningful change threshold in ET.

### MPN-SAF TSS; MPN-10

The development and modification of the MPN-SAF TSS; MPN-10 coincided with the development of the MFSAF versions. Most of the items between the two instruments overlap, but the MPN-SAF TSS; MPN-10 does not use the pain under the ribs (left side item) and includes items for inactivity, concentration problems, fever, and weight loss (Table 1). However, the distinct difference between the two are in the instructions, response options, and recall period leaving the face validity as a fit-for-purpose instrument with major limitations. In the MPN-SAF TSS; MPN-10, the instructions and recall period for the fatigue item differs from the other 9 items (see Table 1). The instructions for the fatigue scale indicates which rating is considered favorable or not favorable which could create a bias in the patient’s rating. The response option scale is the same (0 to 10) across items, but the description for the 0 rating is different between fatigue (0 = no fatigue) and the other 9 items (0 = absent). Additionally, the fever item presents a cutoff threshold (fever > 37.8◦C or >100◦F) making it difficult to assess on a 0 to 10 scale asking about difficulty over the past week. Although there is supportive psychometric evidence for the TSS in the MPN-SAF TSS; MPN-10 instrument [13] (Table 3) and its comparability to the MFSAF v4.0 [20, 23], the variations in the instructions, response options, and recall period the interpretability concerns of the TSS reduce its suitability as a fit-for-purpose instrument.

### PROMIS-Fatigue

The PROMIS Fatigue 7a is one of the PRO measures developed by the National Institutes of Health as part of the larger PROMIS program [16]. The PROMIS Fatigue item banks assess a range of self-reported symptoms, ranging from mild subjective feelings of tiredness to an overwhelming, debilitating, and sustained sense of exhaustion that likely decreases one’s ability to execute daily activities and function normally in family or social roles. The original adult short form was constructed by a domain team with a focus on representing the range of the trait, and also representing the content of the item bank. Domain experts reviewed the short form to give input on the relevance of each item [16], however, the PROMIS Fatigue was not developed with patient input into the item development and has not been debriefed in the ET population.

Although psychometric evaluation has been published in multiple populations, there are no psychometric validation studies published using the PROMIS Fatigue 7a in ET. In MPN populations, the PROMIS Fatigue 7a showed ability to detect change in a randomized, double-blind, placebo-controlled trial of ruxolitinib patients with primary myelofibrosis, post-PV MF, or post-ET MF. [24] Statistically significant improvements with ruxolitinib treatment were identified at Week 24 relative to baseline and placebo in PROMIS Fatigue 7a scale score (*p* < 0.05), demonstrating capacity of the measure to detect change over time. The PROMIS Fatigue 7a showed high internal consistency (Cronbach’s α = 0.84) among myalgic encephalomyelitis/chronic fatigue syndrome participants [26].

Some evidence to support the meaningful change threshold for the PROMIS Fatigue 7a has been identified in the literature [25], but not specifically in ET or MPN more broadly. One study was conducted to estimate meaningful change thresholds for the PROMIS Fatigue 7a in Rheumatoid Arthritis with *N* = 521 patients. Results showed that at 6-months, 7.9% of patients reported their fatigue as “a little better” from baseline (mean PROMIS Fatigue 7a change [SD]: −2.6 [4.8]) with 22.8% reporting their fatigue symptoms being “a little worse” (mean PROMIS Fatigue 7a change 1.7 [5.6]); similar results were reported at 12-months. The estimated MCT range was 1- to 2-points [25].

### EORTC QLQ-C30

The EORTC QLQ-C30 does not have published qualitative studies with ET patients nor has it been psychometrically validated in the ET population specifically. However, the instrument has undergone evaluation in broader oncology populations and demonstrates good psychometric properties. The EORTC QLQ-C30 includes 30 items, the 2 QoL items are scored on a 7-point 1 to 7 scale and all others scored on a 4-point 1 to 4 scale. Recent work has reviewed the content validity of the EORTC QLQ-C30 with regard to oncology populations and current guidance from regulatory authorities [27]. Cognitive debriefing interviews were conducted with 113 cancer patients (including breast, lung, prostate, colorectal, and other cancers) across several disease sites. Cognitive debriefing demonstrated that the most frequently cited spontaneously elicited concepts were already covered in the measure. Item relevance was high across all subgroups and item understanding was at least 90% for all items [27].

The EORTC QLQ-C30 was administered before and once during treatment to 305 lung cancer patients in 13 countries. The data supported the scale structure with the exception of the role functioning scale [14]. The physical functioning scale showed sufficient internal consistency (pretreatment Cronbach α = 0.68 and on-treatment Cronbach α = 0.71). Validity was demonstrated through scale correlations. Physical functioning correlated highly with role functioning (Pearson’s *r* = 0.62) and fatigue (*r* = −0.63) (negative values were noted as an artifact of scaling. Tests of known-groups validity also yielded consistent results with physical functioning being statistically significant in both the pre- and on-treatment groups. Repeated measures analysis of variance showed responsiveness to change in health status on the physical functioning scale (*p* = <0.001). In direct comparison between the EORTC QLC-Q30 and the FACT-G, correlations across 9 studies have been moderate (0.30–0.49) to high (≥0.50) [27].

The EORTC QLQ-C30 showed ability to detect change in a randomized, double-blind, placebo-controlled trial of ruxolitinib patients with primary MF, post-PV MF, or post-ET MF [24]. Significant improvements with ruxolitinib treatment were identified at Week 24 relative to baseline and placebo in all EORTC QLQ-C30 scale scores except for cognitive functioning, constipation, and financial difficulties (*p* < 0.05), demonstrating capacity of the measure to detect change over time [24] in an MPN population, though not specifically ET.

### BFI

The BFI is a brief screening tool designed to assess the severity and impact of fatigue on daily functioning. It’s simple, easy-to-understand language and limited administration time (~10 minutes) make it an easy-to-use measure for older adults [17]. The BFI has been validated in multiple languages and used in other samples including individual with rheumatoid arthritis [30], however, the BFI has not been psychometrically validated in patients with ET. During development of the MFSAF and the original MPN-SAF the BFI was used as the fatigue assessment, however no qualitative evidence for conceptual relevance or quantitative evidence on psychometric properties was published. Eventually only one item from the BFI was retained within the MFSAF v4.0 and MPN-SAF TSS; MPN-10 instruments, thus, the BFI instrument as a whole is not recommended for use in the ET population without further development of the context for use.

## Discussion

The targeted literature review and subsequent conceptual framework identified fatigue as the most often reported symptom for ET whilst physical functioning and activities of daily living were proximal impacts. Distal impacts of ET included emotional, cognitive difficulties, and general life concepts such as financial burden, work productivity and activity, and family relationships. From more than 40 COAs identified in the literature, 5 PROs were identified as most suitable measures to analyze further for conceptual coverage based on the proposed conceptual framework and strength of psychometric evidence in the ET population: MFSAF v4.0, MPN-SAF TSS; MPN-10, PROMIS Fatigue 7a, EORTC QLQ-C30, and the BFI. Although the MPN-SAF TSS; MPN-10 has slightly more conceptual coverage than the MFSAF v4.0, there are limitations with its use in clinical studies due to the instruction format and response option variations between items (Table 1) that can affect respondent understanding and interpretability of the score or changes in the score.

The MFSAF v4.0 content development history included MPN patients [31, 32] and the present analysis demonstrated strong symptom conceptual coverage with the ET conceptual model, but is limited for proximal or distal impacts. The psychometric evidence is good and has included MPN samples which contain ET patients [20–22], but psychometric evidence has not been reported separately for ET. The MFSAF v4.0 uses consistent instructions to rate severity and provides the same response scale for each item. Validation of the MFSAF v4.0 included confirmation that it correlates strongly with the scores from the MPN-10, MPN-SAF, and MF-SAF v2.0 instruments, indicating a high level of comparability to the versions of instruments used in ET populations. [23] The MFSAF v4.0 has undergone extensive reviews with regulatory authorities and is currently the recommended PRO for MPN clinical studies in clinical guidelines and the FDA.

The EORTC QLQ-C30 is a strong candidate PRO to use in ET populations due to its conceptual coverage with the ET patient model proximal and distal impacts (Table 2). Strong psychometric properties have been demonstrated in oncology trials and the instrument is used in clinical studies [14, 24, 27], however, further validation in the ET population, particularly with reference to meaningful changes, is warranted. The PROMIS Fatigue 7a is a good candidate PRO for assessing fatigue impacts in ET and is a stronger candidate for use versus the BFI, due to its fit with current guidelines for COA measures in clinical trials [33, 34], including its history of use as an endpoint in clinical trials. Limitations of the PROMIS Fatigue 7a is that the item development did not include patient feedback and it has not been debriefed with ET patients, thus further debriefing within the ET population would be warranted.

This review of the evidence on COA measures in ET is limited to the information in the published literature. The conceptual framework proposed is based on this literature and could be further strengthened by additional interviews with ET patients specifically. However, the articles reviewed were published peer reviewed studies of a reasonable standard, and there was a specific effort to search for qualitative manuscripts. A strength of the literature review approach is that it provides solid results to identify the appropriate instruments used to measure outcomes in ET. Because this was not a systematic literature review, there may have been fewer articles missed.

## Conclusions

This review and gap analysis has provided information to support the content relevance as well as measurement reliability and validity of the MFSAF v4.0, EORTC QLQ-C30, and PROMIS Fatigue 7a as PRO measures to derive symptom and proximal impact endpoints in patients with ET in a clinical trial.

The development of the content validity and psychometric evidence supporting their use in the ET population is in early stages as a fit for purpose instrument in clinical trials, but they are sufficient compared to other existing COAs. Additional research is needed to generate further content and psychometric validity gaps and meaningful change estimates in symptom focused instruments and PROs that measure proximal and distal impacts in the ET population is warranted.

## Electronic supplementary material

Below is the link to the electronic supplementary material.

Supplementary Material 1

Supplementary Material 2

Supplementary Material 3

## Data Availability

Supplemental files include the search criteria used for this analysis.
