# Supplementary material for: Targeted literature review to understand the patient experience of essential thrombocythemia and evaluate existing clinical outcome assessment measures
Source: J Patient Rep Outcomes. 2026 Oct 1;10:183. doi: 10.1186/s41687-026-01048-7 (PMC13650201; doi:10.1186/s41687-026-01048-7)
Supplement: Supplementary file 1 [file 41687_2026_1048_MOESM1_ESM.docx]

**Table A. Search Terms and Methodology for Qualitative and Descriptive Studies in ET**

| Condition | Assessment | Concept |
| --- | --- | --- |
|  | **AND** | **AND** |
| exp essential thrombocythemia/ OR (thrombocythemia* AND (blood)* OR myeloproliferative neoplasm* OR myeloproliferative disorder)).ti,ab,kw,hw,tw | exp patient-reported outcome/ OR exp Patient Reported Outcome Measures/ OR exp "Surveys and Questionnaires"/ OR exp quality of life/ OR exp health survey/ OR exp questionnaire/ OR exp self report/ OR exp rating scale/ OR exp functional status assessment/ OR exp functional assessment/ OR exp self evaluation/ OR symptoms/ OR exp symptom assessment/ OR ((Patient (report* OR outcome* OR experience*)) OR instrument* OR measure* OR diar*).ti,ab,kw,hw,tw. | (qualitative OR interview* OR focus group* OR survey OR prospective stud* OR clinical trial* OR observational stud* OR narrative* OR (patient (opinion* OR perspective* OR experience*)) OR psychometric* OR "context of use" OR (valid* AND (construct OR content. ti,ab,kw,hw,tw. |

ET = essential thrombocythemia

Search fields: title, abstract or keyword

Limitations applied: Year: 2012-current; Subject: Human; Language: English only

Note: * OVID Wildcard used to retrieve unlimited suffix variations. MPN was removed due to returning a large number of articles not related to the research question.
