# Supplementary material for: Targeted literature review to understand the patient experience of essential thrombocythemia and evaluate existing clinical outcome assessment measures
Source: J Patient Rep Outcomes. 2026 Oct 1;10:183. doi: 10.1186/s41687-026-01048-7 (PMC13650201; doi:10.1186/s41687-026-01048-7)
Supplement: Supplementary file 2 [file 41687_2026_1048_MOESM2_ESM.docx]

**Table B. Search Terms and Methodology for COA Measures in ET**

| Condition | Assessment | Concept |
| --- | --- | --- |
|  | **AND** | **AND** |
| exp essential thrombocythemia/ | (Self* OR  Patient* OR Clinician*) ADJ1  (Rated OR Reported)  OR  (Self* OR  Patient* OR  Clinician*OR  Observer* OR  PRO OR  ClinRO OR  ObsRO)  ADJ2  (Questionnaire* OR  Instrument* OR  Rating* OR  Scale* OR  Diary OR  Diaries OR  Measure*) OR  constitutional OR symptom* OR lifestyle OR burden OR sign OR signs | (qualitative OR interview* OR focus group* OR survey OR prospective stud* OR clinical trial* OR observational stud* OR narrative* OR prevalence OR incidence OR patient opinion* OR patient perspective* OR patient experience* OR psychometric* OR “context of use” OR (valid* AND construct) OR (valid* AND content) OR HRQoL OR QOL OR Health related quality of life |

ClinRO = clinician-reported outcome; COA = clinical outcome assessment; ET = essential thrombocythemia; ObsRO = observer reported outcome; PRO = patient-reported outcome

ADJ: searches for the terms adjacent to or within 1 or 2 words of each other

Search fields: title, abstract or keyword

Limitations applied: Year: 2012-current; Subject: Human; Language: English only

Note: * OVID Wildcard used to retrieve unlimited suffix variations. MPN was removed due to returning a large number of articles not related to the research question.
