# Supplementary material for: Targeted literature review to understand the patient experience of essential thrombocythemia and evaluate existing clinical outcome assessment measures
Source: J Patient Rep Outcomes. 2026 Oct 1;10:183. doi: 10.1186/s41687-026-01048-7 (PMC13650201; doi:10.1186/s41687-026-01048-7)
Supplement: Supplementary file 3 [file 41687_2026_1048_MOESM3_ESM.docx]

| **Palandri 2018^1^** | | | | | | | | | | | | | | |
| --- | --- | --- | --- | --- | --- | --- | --- | --- | --- | --- | --- | --- | --- | --- |
| **Population** | | **Methods** | | | **Symptom (%)*** | | | | | **Impacts (%)*** | | | | |
| - Italy - Primary or post-Essential Thrombocythemia/post-Polycythemia Vera MF patients - (Primary myelofibrosis – 44%; Post-ET - 21%; Post-PV – 19%; No answer – 16% ) - Narratives: N= 215 patients; N= 62 caregivers - Questionnaires: N= 287 patients; N=98 caregivers - Median age 65 years (range 27-89) - 55% male | | - Qualitative - Narratives and semi- structured questionnaires | | | Fatigue (70%) | | | | | Mobility   - Walking (49%) - Daytime confined to bed | | | | |
|  |  |  |  |  | Splenomegaly (68%) | | | | |  |  |  |  |  |
|  |  |  |  |  | Systemic symptoms (63%)   - insomnia, - bone pain, - itching, - loss of appetite, - stress, - muscle aches | | | | |  |  |  |  |  |
|  |  |  |  |  |  |  |  |  |  | Activities of daily living   - Outdoor activities (49%) - Domestic activities (23%) - Self care (6%) | | | | |
|  |  |  |  |  | Constitutional symptoms (49%)   - sweating, - weight loss, - fever | | | | |  |  |  |  |  |
|  |  |  |  |  |  |  |  |  |  | Emotional Impact   - Fear (53%) - Sorrow (23%) - Relief (11%) - Resignation (7%) - Anger (6%) | | | | |
|  |  |  |  |  |  |  |  |  |  | Economic Impact   - Loss of earnings (22%) | | | | |
|  |  |  |  |  |  |  |  |  |  | Reliance on caregiver (63%) | | | | |
| * Symptoms and impacts were recorded in patient narratives only (N=215), percentages have been reported where available | | | | | | | | | | | | | | |
|  | | | | | | | | | | | | | | |
| **Ritchie 2022^2^** | | | | | | | | | | | | | | |
| **Population** | **Methods** | | **Symptoms and Impacts** | | | | | | | | | **Mean (SD) individual symptom score** | | |
| United States  MF and ET patients  **Results reported here for ET patients only**   - N=801 - Median age 70 (range 0-37) - Male - 32.2% - White – 89.6% | The Myelofibrosis and Essential Thrombocythemia Observational Study (MOST)  PRO data collected at enrollment, usual care visits over 36 month observation period   - MPN-SAF - EORTC QLQ-C30 - WPAI-SHP | | **MPN-SAF enrollment n=772** | | | | | | | | |  | | |
|  |  |  | Fatigue | | | | | | | | | 3.4 (2.7) | | |
|  |  |  | Early satiety | | | | | | | | | 2.3 (2.7) | | |
|  |  |  | Inactivity | | | | | | | | | 2.3 (2.8) | | |
|  |  |  | Numbness/tingling | | | | | | | | | 2.2 (3.0) | | |
|  |  |  | Concentration problems | | | | | | | | | 1.9 (2.6) | | |
|  |  |  | Bone pain | | | | | | | | | 1.6 (2.7) | | |
|  |  |  | Abdominal discomfort | | | | | | | | | 1.6 (2.5) | | |
|  |  |  | Night sweats | | | | | | | | | 1.5 (2.6) | | |
|  |  |  | Itching | | | | | | | | | 1.5 (2.4) | | |
|  |  |  | Weight loss | | | | | | | | | 0.7 (1.8) | | |
|  |  |  | Fever (>100F) | | | | | | | | | 0.3 (1.0) | | |
|  |  |  | **EORTC QLQ-C30 enrollment n= 798** | | | | | | | | |  | | |
|  |  |  | Fatigue | | | | | | | | | 29.5 (25.8) | | |
|  |  |  | Insomnia | | | | | | | | | 28.5 (30.6) | | |
|  |  |  | Pain | | | | | | | | | 22.1 (27.8) | | |
|  |  |  | Dyspnea | | | | | | | | | 17.1 (25.4) | | |
|  |  |  | Constipation | | | | | | | | | 15.6 (25.2) | | |
|  |  |  | Appetite loss | | | | | | | | | 12.1 (23.1) | | |
|  |  |  | Financial difficulties | | | | | | | | | 11.7 (24.6) | | |
|  |  |  | Diarrhea | | | | | | | | | 9.4 (19.3) | | |
|  |  |  | Nausea and vomiting | | | | | | | | | 5.4 (12.9) | | |
|  |  |  | **WPAI enrollment n=791** | | | | | | | | |  | | |
|  |  |  | Currently employed 231 (29.2%) | | | | | | | | |  | | |
|  |  |  | Absenteeism | | | | | | | | | 2.9 (10.0) | | |
|  |  |  | Presenteesim | | | | | | | | | 11.5 (21.2) | | |
|  |  |  | Overall work productivity loss | | | | | | | | | 11.5 (21.2) | | |
|  |  |  | Activity Impairment | | | | | | | | | 18.3 (25.5) | | |
|  | | | | | | | | | | | | | | |
| **Byun 2022^3^ Landmark Health Survey** | | | | | | | | | | | | | | |
| **Population** | **MPN-SAF symptoms within the last 12 months (patient reported)** | | **Average Score**  **ET Cohort, Mean (SD)** | | | | | | | | | **Incidence**  **ET Cohort, n(%)** | | |
| Korea  N=105  MF n=39; PV n= 25; ET n=41  **Results reported here for ET patients only; n=41**  Age at MPN diagnosis  55 (29-76)  Male 16 (39.02) | Fatigue or tiredness | | 5.00 + 2.26 | | | | | | | | | 32 (78.05) | | |
|  | Abdominal discomfort | | 3.07 + 2.63 | | | | | | | | | 9 (21.95) | | |
|  | Night sweats | | 1.80 + 2.94 | | | | | | | | | 5 (12.20) | | |
|  | Filling up quickly when eating | | 2.75 + 3.02 | | | | | | | | | 4 (9.76) | | |
|  | Bone pain | | 1.67 + 1.97 | | | | | | | | | 3 (7.32) | | |
|  | Inactivity | | 3.77 + 3.14 | | | | | | | | | 6 (14.63) | | |
|  | Itching (pruritis) | | 2.70 + 1.87 | | | | | | | | | 12 (29.27) | | |
|  | Unintentional weight loss | | 1.58 + 3.09 | | | | | | | | | 3 (7.32) | | |
|  | Problems with concentration | | 2.81 + 2.40 | | | | | | | | | 7 (17.07) | | |
|  | Fever | | 1.30 + 1.95 | | | | | | | | | 3 (7.32) | | |
|  | MPN-SAF TSS  Mean + SD  Median (range) | | 13.59 + 12.14  9.00 (0.00-53.00) | | | | | | | | | | | |
|  | Values are presented as mean + standard deviation or number (%) | | | | | | | | | | | | | |
|  | | | | | | | | | | | | | | |
| **Yacoub 2021^4^** | | | | | | | | | | | | | | |
| **Population** | **Methods** | | | **Signs and Symptoms of ET Documented by Health Care Providers at Enrollment** | | | | | | | | | | |
|  |  |  |  |  | | | | | **Incidence** | | | | | |
| - United States - 1207 ET patients in this analysis - Median age 70 (19-65 yrs) and 65% female at enrollment | - The Myelofibrosis and Essential Thrombocythemia Observational Study (MOST) - Clinical characteristics of patients with ET at the time of diagnosis and at the time of enrollment in MOST. | | | - Vasomotor | | | | |  | | | | | |
|  |  |  |  | - Headache | | | | | 6.1% | | | | | |
|  |  |  |  | - Lightheadedness | | | | | 2.9% | | | | | |
|  |  |  |  | - Transient visual disturbances | | | | | 1.3% | | | | | |
|  |  |  |  | - Atypical chest pain | | | | | 1.2% | | | | | |
|  |  |  |  | - Acral paresthesia | | | | | 0.9% | | | | | |
|  |  |  |  | - Erythromelalgia | | | | | 0.2% | | | | | |
|  |  |  |  | - Livedo reticularis | | | | | 0% | | | | | |
|  |  |  |  | - Constitutional | | | | |  | | | | | |
|  |  |  |  | - Fatigue | | | | | 25.4% | | | | | |
|  |  |  |  | - Night sweats | | | | | 4.2% | | | | | |
|  |  |  |  | - Weight loss | | | | | 2.2% | | | | | |
|  |  |  |  | - Fever | | | | | 0.4% | | | | | |
|  |  |  |  | - Spleen-associated | | | | |  | | | | | |
|  |  |  |  | - Abdominal pain | | | | | 1.7% | | | | | |
|  |  |  |  | - Early satiety | | | | | 1.6% | | | | | |
|  |  |  |  | - Pruritus | | | | |  | | | | | |
|  |  |  |  | - Pruritis | | | | | 3.3% | | | | | |
|  | | | | | | | | | | | | | | |
| **Harrison 2017^5^** | | | | | | | | | | | | | | |
| **Population** | **Methods** | | | **Symptoms experienced by ET patients in the past 12 months** | | | | | **Impacts reported by all patient cohorts^2^** | | | | | |
| Six countries   - Australia - Canada - Germany - Italy - Japan - UK   MPN patients N= 699   - n= 174 MF - n= 223 PV - n= 302 ET   **ET patients**   - Female 68%   Mean age 54.9 years | MPN Landmark Survey   - Includes questions adapted from MPN-SAF and EORTC QLQ-C30 - Multi-country, cross sectional survey of patients diagnosed with MPN and treating physicians - Patient and physician surveys - April 2016 to October 2016 | | | Fatigue | | | | 64% | Physical hardship | | | | | 64% |
|  |  |  |  | Dizziness/vertigo/  lightheadedness | | | | 33% | Emotional hardship | | | | | 67% |
|  |  |  |  | Night sweats | | | | 31% | Anxious | | | | | 78% |
|  |  |  |  | Bruising | | | | 30% | Worry | | | | | 89% |
|  |  |  |  | Difficulty sleeping | | | | 28% | Depressed | | | | | 61% |
|  |  |  |  | Depression or sad mood | | | | 26% | Financial hardship | | | | | 46% |
|  |  |  |  | Numbness/tingling in hands and feet | | | | 24% | ADLs | | | | | 76% |
|  |  |  |  | Pruritus | | | | 24% | Relationships caregiver | | | | | 74% |
|  |  |  |  | Loss of concentration | | | | 23% | Relationships family or social life | | | | | 70% |
|  |  |  |  | Shortness of breath | | | | 19% | Limit activities due to pain/discomfort | | | | | 64% |
|  |  |  |  | Abdominal discomfort | | | | 15% | Work | | | | | |
|  |  |  |  | Weakness | | | | 14% | Voluntarily left work | | | | | 9% |
|  |  |  |  |  | | | | | Early retirement | | | | | 8% |
|  |  |  |  |  |  |  |  |  | Disability allowance | | | | | 7% |
|  |  |  |  |  |  |  |  |  | Moved to a lower paying job | | | | | 5% |
|  |  |  |  |  |  |  |  |  | Involuntary loss of work | | | | | 2% |
|  | | | | | | | | | | | | | | |
| **Website** | **Population** | | | **Common symptoms reported by people with ET** | | | | | | | | | | |
| **Patients Like Me^6^** | - Essential thrombocythemia - People self-reporting their common symptoms on an online website | | |  |  |  |  |  |  |  |  |  |  |  |
|  |  |  |  |  | | Severe | Moderate | | | | Mild | | None | |
|  |  |  |  | Fatigue | | 22 (22%) | 46 (47%) | | | | 19 (19%) | | 10 (10%) | |
|  |  |  |  | Stress | | 10 (10%) | 11 (29%) | | | | 18 (48%) | | 4 (10%) | |
|  |  |  |  | Anxious mood | | 9 (9%) | 32 (32%) | | | | 30 (31%) | | 24 (25%) | |
|  |  |  |  | Pain | | 9 (9%) | 31 (32%) | | | | 28 (29%) | | 27 (25%) | |
|  |  |  |  | Depressed mood | | 5 (5%) | 25 (26%) | | | | 34 (35%) | | 31 (32%) | |

1. Palandri, F., Benevolo, G., Iurlo, A., Abruzzese, E., Carella, A. M., Paoli, C., ... & Reale, L. (2018). Life for patients with myelofibrosis: the physical, emotional and financial impact, collected using narrative medicine—Results from the Italian ‘Back to Life’project. Quality of Life Research, 27, 1545-1554.
2. Ritchie, E., Al-Janadi, A., Kessler, C., Scherber, R., Kalafut, T., Ren, H., & Mesa, R. (2022). Patient-reported outcomes of patients with myelofibrosis or essential thrombocythemia enrolled in the MOST study. Leukemia & Lymphoma, 63(13), 3138-3153.
3. Byun, J. M., Bang, S. M., Choi, E. J., Eom, K. S., Jung, C. W., Kim, H. S., ... & Choi, C. W. (2022). How myeloproliferative neoplasms patients’ experience and expectations differ from physicians’: the international MPN Landmark survey. The Korean Journal of Internal Medicine, 37(2), 444.
4. Yacoub, A., Lyons, R., Verstovsek, S., Shao, R., Chu, D. T., Agrawal, A., ... & Mascarenhas, J. (2021). Disease and clinical characteristics of patients with a clinical diagnosis of essential thrombocythemia enrolled in the MOST study. Clinical Lymphoma Myeloma and Leukemia, 21(7), 461-469.
5. Harrison, C. N., Koschmieder, S., Foltz, L., Guglielmelli, P., Flindt, T., Koehler, M., ... & Mesa, R. A. (2017). The impact of myeloproliferative neoplasms (MPNs) on patient quality of life and productivity: results from the international MPN Landmark survey. Annals of hematology, 96, 1653-1665.
6. Patients Like Me website available at: https://www.patientslikeme.com/conditions/essential-thrombocythemia
